# Application of Hydrophilic Polymers to the Preparation of Prolonged-Release Minitablets with Bromhexine Hydrochloride and Bisoprolol Fumarate

**DOI:** 10.3390/pharmaceutics16091153

**Published:** 2024-08-30

**Authors:** Agata Grzejdziak, Witold Brniak, Olaf Lengier, Justyna Anna Żarek, Dziyana Hliabovich, Aleksander Mendyk

**Affiliations:** 1 Students Scientific Group of Pharmaceutical Technology, Faculty of Pharmacy, Jagiellonian University Medical College, Medyczna 9, 30-688 Kraków, Poland; 2 Department of Pharmaceutical Technology and Biopharmaceutics, Faculty of Pharmacy, Jagiellonian University Medical College, Medyczna 9, 30-688 Kraków, Poland

**Keywords:** minitablets, prolonged release, dissolution kinetics, polymers, bromhexine hydrochloride, bisoprolol fumarate, direct compression

## Abstract

Minitablets have been extensively studied in recent years as a convenient pediatric form because they allow successful administration even in very young children. Their advantages include easy dose adjustment by multiplication of single units as well as the possibility of drug release modification by coating or forming matrix systems. The aim of this study was to demonstrate the possibility of the formulation of prolonged-release minitablets with bromhexine hydrochloride (BHX) and bisoprolol fumarate (BFM) dedicated to pediatric patients. Minitablets with 3 mm diameter and 15 mg mass, containing 1 mg of active substance in 1 unit, were prepared by direct compression with hydroxypropyl methylcellulose (HPMC) of different grades, methylcellulose, sodium alginate, or polyvinyl alcohol (PVA) as a sustained-release polymer. Different amounts of polymers and different compression forces were evaluated. Analysis of minitablets included their uniformity, hardness, and dissolution tests. The kinetics of drug substance release were analyzed with dedicated software. The prepared minitablets met the pharmacopeial requirements with respect to the uniformity of mass and content. The compressibility of BFM was significantly better than that of BHX, yet all minitablets had good mechanical properties. Dissolution studies showed a strong relationship between the type of polymer and its amount in the mass of a tablet and the dissolution rate. Prolonged release of up to 8 h was achieved when HPMC of 4000 cP viscosity was used in the amount of 30% to 80%. Sodium alginate in the amount of 50% was also effective in prolonging dissolution, but PVA was much less effective. Studies on the release kinetics showed that dissolution from prolonged-release minitablets with BHX fit the best to Hopfenberg or Hixson–Crowell models, while in the case of BFM, the best fit was found for Hopfenberg or Korsmeyer–Peppas models.

## 1. Introduction

The oral route remains the most common method of drug administration due to the ease of self-administration by patients or caregivers, the highest acceptability, and the possibility of controlling and modifying the release of the active pharmaceutical ingredient (API) [1,2]. Solid oral dosage forms, particularly tablets, are the most popular kind of formulation. Their advantages include dosage accuracy, stability during storage, resistance to handling and transportation, and many more. The major drawback of tablet administration is the frequent problem of swallowing, especially encountered in pediatric and geriatric populations [3]. One of the solutions to this problem is formulation of minitablets [4]. They are a solid dosage form with a typical diameter of 1–3 mm and a weight of approximately 5–25 mg. Due to their small size, they can be swallowed much easier or administered with liquid or soft foods [5,6,7,8].

One of the most popular trends in the development of dosage forms, intended to improve patient compliance, is the reduction in the frequency of dosing by modification of the rate of drug substance release. Prolonged- or sustained-release preparations by definition have a reduced rate of API release and allow the possibility of maintaining a constant blood level of the drug substance for 8–12 h or even longer (e.g., 24 h) [9]. These dosage forms also offer fewer fluctuations in the plasma level of a drug and a lower maximum concentration directly after administration, which can lead to fewer adverse effects [10]. The technological development of extended-release drug forms focuses particularly on substances with a short half-life, ranging from 1 to 6 h [11,12]. It is not recommended to prepare a prolonged-release drug form for substances that are absorbed only in the upper gastrointestinal tract or require a washout period during standard therapy and for those drugs that are used in doses greater than 500 mg. Also excluded are APIs, whose absorption cannot be limited by their dissolution rate [12]. There are numerous technologies used to slow down the release of the drug substance, e.g., coating the semi-finished or finished product with a functional polymer, incorporation of drug substances into matrices that slowly dissolve or erode, complexation with substances that modify solubility, bonding with ion exchangers, modification of the crystallization process, and chemical modification of the drug substance to change its solubility (esterification or salt formation) [12].

The great possibilities in modifying API release come from its incorporation into a functional matrix. The simplest way is to directly compress bulk or granulated API with a mixture of excipients using a tablet press. The tablet matrix may be hydrophilic, lipophilic, or insoluble in the digestive tract. Tablets made of a hydrophilic matrix are produced mainly for APIs that are poorly soluble in water. In this case, water-soluble polymers are used, which swell in the gastrointestinal tract, creating a high-viscosity hydrogel. These compounds may differ in parameters such as molecular weight, degree of swelling, or viscosity of the hydrogel that forms [13]. The hydrogel layer controls the release rate of the drug substance from the matrix. Water gradually wets the tablet and penetrates into its core, and the dissolved active substance diffuses through a layer of viscous gel that limits its release. This wetted tablet also erodes, and its surface gradually decreases, resulting in a smaller release surface and a slower release process as the tablet moves through the gastrointestinal tract. The balance between erosion rate and changes in the thickness of the gel layer allow stabilization of the API release within a particular time and release with a constant speed. An example of a hydrophilic polymer is hydroxypropylmethylcellulose (HPMC), which is a biodegradable ester derivative of cellulose [14,15]. It is compatible with most APIs and excipients and is odorless, flavorless, transparent, relatively inexpensive, and readily available. HPMC has a linear structure of glucose molecules and is non-ionic [15,16]. Aqueous solutions of HPMC exhibit a sol–gel transition during heating and a reversible gel–sol transition during cooling [17]. Another derivative of cellulose is methylcellulose (MC), available in the form of white powder and insoluble in hot water but forming a colloid in the temperature range 50–70 °C [18,19]. Another example of a hydrophilic polymer is sodium alginate (SA), a natural polysaccharide formed in the cell wall of various species of algae and bacteria. It is nontoxic, biodegradable, and biocompatible. Structurally, alginates are a combination of β-D-mannuronic and α-L-glucuronic acid fractions linked to each other by one to four glycosidic bonds. SA is a white or slightly yellowish powder that is easily soluble in water. In cold water, it can form very viscous solutions and gels when combined with divalent ions [20].

Bisoprolol fumarate (BFM) belongs to a class of medications called beta-blockers [21]. It selectively blocks β1-receptors, preventing their stimulation and limiting the effects of adrenaline or norepinephrine. It has no ability to stabilize cell membranes or intrinsic sympathomimetic activity. Bisoprolol fumarate works by relaxing blood vessels and slowing the heart rate to improve and decrease blood pressure. It is used to treat hypertension, stable angina pectoris, and stable chronic heart failure with impaired left ventricular systolic function (in combination with other drugs) [21]. Bisoprolol fumarate is available on the European market only in the form of immediate-release preparations, and there is no pediatric form available [22]. It is dosed once a day due to a long half-life (approximately 10–12 h).

Bromhexine hydrochloride (BHX) is a well-known expectorant from the group of mucolytics [23]. It is metabolized in the liver to the active compound ambroxol. Bromhexine is indicated in acute and chronic bronchial diseases with excessive mucus secretion. It reduces the content of mucopolysaccharides in mucus, which has an expectorant effect. In addition, it stimulates the synthesis and secretion of surfactant. In this way, it improves the function of the cilia of the respiratory epithelium. It does not disturb the natural cough reflex. The biological half-life of bromhexine is approximately 12 h. Multiple dosage forms with bromhexine are registered, including conventional tablets, orodispersible tablets, syrups, and oral drops. The pediatric dosing regimen requires drug administration three times a day, which can cause problems with acceptability, compliance, and effective therapy. Unfortunately, there is no modified release dosage form containing bromhexine [24].

**Figure 1:**
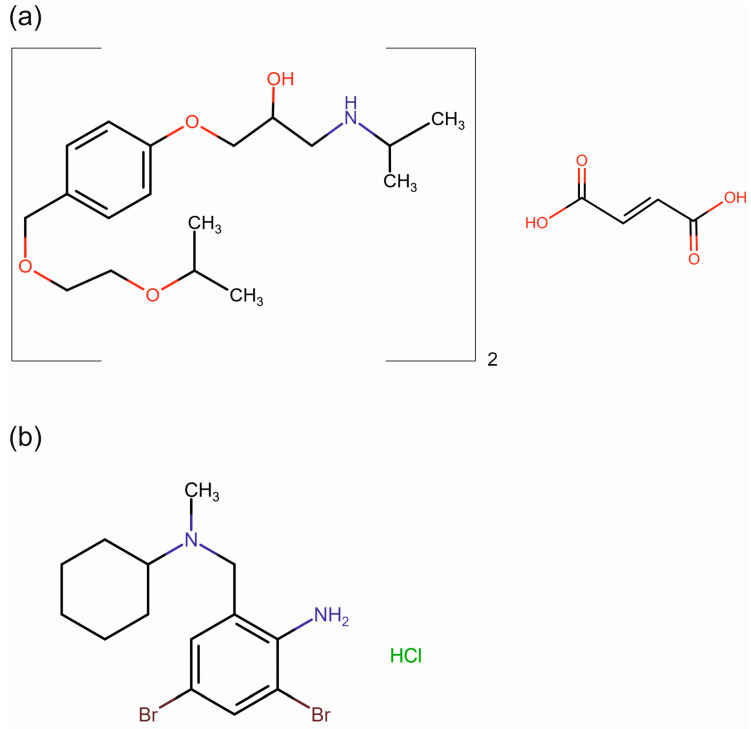
Chemical structure of bisoprolol fumarate (**a**) and bromhexine hydrochloride (**b**).

The chemical structures of bisoprolol fumarate and bromhexine hydrochloride are presented in Figure 1. BFM is highly soluble in water and highly permeable, thus belonging to a class I of the Biopharmaceutical Classification System (BCS). BHX is poorly soluble in water and neutral media, but its solubility in the acidic conditions is much higher. It belongs to BCS class II.

The aim of our study was to assess the possibility of development of extended-release minitablets based on hydrophilic polymers and with selected model drugs used in pediatric therapies. The minitablet formulation is intended to facilitate easy swallowing due to its small size, while the extended form can make administration less frequent, leading to better compliance. Furthermore, the great advantage of prolonged-release minitablets is the lack of risk of releasing the entire dose of the active substance in the event of damage to the coating of a single tablet as well as the fact that the release is independent of the frequency of gastric emptying [25]. The incorporation of a small dose into a single minitablet also allows easy and precise dose adjustment in pediatric patients according to their body weight.

## 2. Materials and Methods

### 2.1. Materials

Two active pharmaceutical ingredients (API) were used for the study: bromhexine hydrochloride (99.4% purity) (BHX) (VenPetrochem, Mumbai, India) and bisoprolol fumarate (99.5% purity) (BFM) (Wuhan ChemNorm Biotech Co., Ltd., Wuhan, China). The other excipients included Metolose 90SH-400 and Metolose 90SH-4000 cP—hydroxypropyl methylcellulose (Shin-Etsu Chemical, Tokyo, Japan); Metolose SM 4000 cP—methylcellulose (Shin-Etsu Chemical, Tokyo, Japan); sodium alginate (Sigma-Aldrich, St. Louis, MO, USA); Parteck SRP 80—polyvinyl alcohol (Merck, Darmstadt, Germany); Flowlac 100—spray-dried lactose (Meggle Pharma, Wasserburg am Inn, Germany); Pearlitol 100SD—D-mannitol (Roquette, Lestrem, France); Vivapur 101—microcrystalline cellulose (JRS Pharma, Holzmühle, Germany); Cab-O-Sil—silicon dioxide (Cabot, Rheinfelden, Germany); and Pruv—sodium stearyl fumarate (JRS Pharma, Holzmühle, Germany). All other ingredients were of analytical grade. The water for the preparation of solutions was purified with the reversed-osmosis Elix Essential 15 UV system (Merck Millipore, Molsheim, France).

### 2.2. Preparation of Minitablets

Preparation of minitablets included three different stages with a focus on three different aspects of this process and its relationship with minitablets properties. In the first one, the effect of composition on the properties of minitablets was studied, with a main focus on the drug release process. In the second stage, the effect of different compression force on the minitablets’ properties was evaluated. The third stage consisted of the characteristic of compression process.

In the first stage of the study, eight series of BHX minitablets and thirteen series of BFM minitablets were prepared using a single-punch tablet press EK0 (Korsch, Berlin, Germany). Each minitablet contained 1 mg each of API and one of the sustained release polymers: sodium alginate, polyvinyl alcohol, hydroxypropylmethylcellulose, or methylcellulose (different grades and amounts). They also contained microcrystalline cellulose, lactose, mannitol, silicon dioxide, and sodium stearyl fumarate (Table 1, Table 2 and Table 3). The minitablets had a diameter of 3 mm and a mass of 15 mg. The names of the formulations were encoded with the name of the active substance, the amount of polymer used, and its type: BH—bromhexine hydrochloride; BF—bisoprolol fumarate; A—Metolose 90SH-400; B—Metolose 90SH-4000; C—Metolose SM-4000; D—sodium alginate; E—polyvinyl alcohol; m—D-mannitol.

In the second stage of the study, three series of minitablets with the same composition as formulation BF_60B were prepared with the single-punch tablet press EK0 (Korsch, Berlin, Germany). The difference between these series came from three different compression forces used to obtain tablets with the following target hardness: lower than 1.5 kp, from 1.8 to 2.2 kp, and from 3.0 to 4.0 kp. These minitablets were used to study the effect of compression force on the dissolution profile.

### 2.3. Measurement of Compression Parameters

To assess the compressibility of tablet masses containing hydroxypropyl methylcellulose (HPMC), the tableting process was simulated using the EZ-SX texture analyzer (Shimadzu, Suzhou, China) with four different compression forces: 200 N, 300 N, 400 N, and 450 N. The test was carried out for tablet masses containing 50% and 60% of HPMC (formulations BH_50B and BF_60B). To simulate the tableting process, the special probe was prepared, designed, and manufactured at the Department of Pharmaceutical Technology and Biopharmaceutics. It consisted of a single minitablet punch and die (with a diameter of 3 mm diameter) adapted from EK0 eccentric tablet press. The punch was attached to the measuring head, which had a maximum load of 500 N. The die was mounted on the flat surface of the table.

The compression test was performed six times for each formulation using 15 mg of tablet mass weighed on the analytical scale MS105DU (Mettler Toledo, Greifensee, Switzerland) and transferred with a spatula to the tablet die. The mass was compressed by a single punch moving downward with a constant speed of 10 mm/min. During the compression of the minitablets, the force acting on the tablet mass expressed as a function of time and displacement of the punch were recorded. The values of the total compression energy (E_t_) and its component, called plastic energy (E_p_), were calculated using the Trapezium X software v. 1.5.2 (Shimadzu, Kyoto, Japan). The second component of total compression energy, i.e., the elastic energy of compression (E_e_), was calculated with an Excel spreadsheet (Microsoft for Windows 365 MSO v.2404) as the difference between the total compression energy and the plastic energy (E_e_ = E_t_ − E_p_). Furthermore, the ratio of the elastic energy value to the total energy of compression was calculated. Arithmetic means and standard deviations were calculated for each series (N = 6).

### 2.4. The Resistance to Crushing (Hardness) Test

The hardness of the minitablets from each prepared batch was measured using an EZ-SX (Shimadzu, Suzhou, China) texture analyzer equipped with a flat cylindrical probe of 10 mm diameter and a 500 N measuring head. Each tablet was placed on its side on the measuring table of the apparatus, while the probe moved at a constant speed of 10 mm/min. The test was finished when the probe detected a crack in the side surface of the minitablet (a sudden drop in resistance force during measurement). The device recorded the value of the maximum force needed to break the tablet. The test was repeated for six tablets from each series. The arithmetic mean and standard deviations were calculated.

### 2.5. Dissolution Studies

Dissolution studies for minitablets were performed using a type II pharmacopeial dissolution apparatus (paddle apparatus) SR8Plus (Hanson Research Corporation, Chatsworth, CA, USA). The test was carried out according to the monograph contained in the United States Pharmacopoeia [26] in 900 mL of distilled water as a medium for minitablets with BFM and in 500 mL of 0.1 mol/L hydrochloric acid solution (pH = 1.2) for series with BHX. The temperature of the medium was maintained at 37 °C, and the paddle rotation speed was 75 rpm. Ten minitablets were placed in each vessel of the apparatus, which resulted in the examination of a total of 30 minitablets from each series. Samples with a volume of 5 mL were collected after 15 min, 30 min, 45 min, and then 1 h, 2 h, 4 h, 8 h, and 12 h from the beginning of the study. The volume of the sample was automatically refilled with the same amount of the medium, which allowed to maintain its constant volume during the test. The content of BHX and BFM was determined in the collected samples using an UV-1900 spectrophotometer (Shimadzu, Kyoto, Japan), measuring the absorbance value at a wavelength of λ = 245 nm for minitablets with BHX and λ = 224 nm for minitablets with BFM. The arithmetic means and standard deviations were calculated for each formulation.

In order to determine the drug release rate, mean dissolution time (MDT) was calculated using following equation [27]:

$$MDT=\sum \frac{t_{i}\times ∆Q_{i}}{Q_{\infty }}$$

where t_i_ is an intermediate time of the intervals of sampling time, ∆Q_i_ the amount of released BHX or BFM in the specified interval of time, and Q_∞_ the maximum amount of BHX or BFM released.

The area under the dissolution curve (AUC) was calculated using Excel spreadsheet (Microsoft for Windows 365 MSO v.2404) based on the linear trapezoidal rule for the dissolution curves in the range from 0 to 12 h.

### 2.6. Uniformity of Minitablets

In order to evaluate the uniformity of the minitablets, three different tests were performed: Uniformity of the mass of single-dose preparations according to the monograph 2.9.5 of the European Pharmacopoeia 11.5 [28], uniformity of content of single-dose preparations (2.9.6) [29], and uniformity of dosage units (2.9.40) [30]. The tests were performed for selected batches of minitablets with BHX (BH_50B) and BFM (BF_20C) using 10 single units in each case.

Ten minitablets were weighed individually using the analytical scale MS105DU (Mettler Toledo, Greifensee, Switzerland). Their average mass and individual deviations were calculated.

In order to evaluate uniformity of content, 10 minitablets were individually dispersed in 50 mL of purified water. After 24 h of shaking on a laboratory shaker KS 130 Basic (IKA, Staufen, Germany) with at speed 400 rpm, the samples were filtered, and the concentration of BFM and BHX was measured with an UV-1900 spectrophotometer as previously described. Due to the higher concentration of APIs in this study, the absorbance of the BFM was measured at λ = 271 nm, which is the value of the maximum of the second (lower) peak on the absorbance curve. Average content values and percentage deviations were calculated.

The uniformity of the dosage units was based on the measurements made for the uniformity of the content study. Individual deviations from the declared values and acceptance values were calculated according to the pharmacopeial monograph [30].

### 2.7. Kinetic Analysis of the Release Process

The kinetics of the dissolution process were analyzed using the dedicated open-source software RKinetDS 1.0 [31]. It performed curve fitting of the dissolution results for the most popular mechanistic and empirical models of the dissolution curves. The analysis was performed for all the prepared formulations of minitablets with BHX and BFM. A minimum of 3 data points without 0 and without plateau, where plateau means less than 5% of drug release between the two following time points (if available), were used for the analysis.

Only a few models were chosen for investigation: 0-, 1-, 2-, and 3-order; Higuchi; Korsmeyer–Peppas; Hopfenberg; and Hixson–Crowell. The best fit with the latter two suggests an erosion-driven drug release mechanism, whereas Higuchi and Korsmeyer–Peppas, when the best-fitting data were found, suggest an erosion-driven mechanism of drug release. The models’ predictability was assessed as root mean squared error (RMSE) between observed and model-predicted values.

### 2.8. Statistical Analysis of Data

Descriptive statistics were calculated using OriginPro 2020b software v.9.7.5.184 or a Microsoft Excel 365 MSO spreadsheet. Statistically significant differences were evaluated based on a one-way analysis of variance (ANOVA) with a post hoc Tukey’s multiple comparison test calculated in the Statistics module of the OriginPro 2020b software. Differences between results were significantly different when the values of *p* were lower than 0.05.

## 3. Results and Discussion

### 3.1. Characteristic of Minitablets

**Figure 2:**
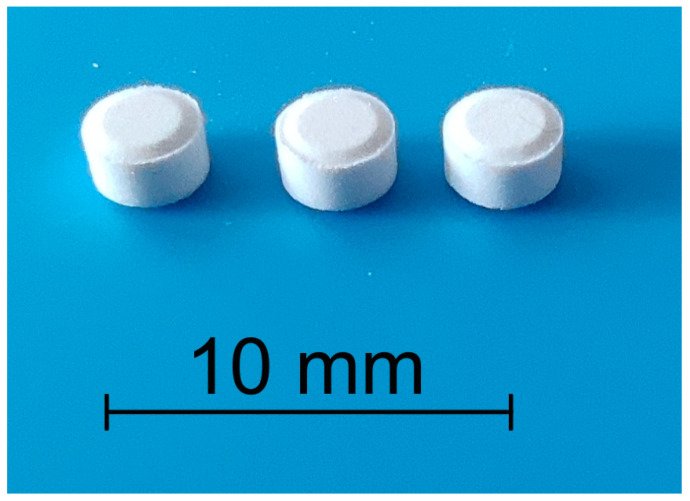
The appearance of the minitablets with bisoprolol fumarate (BFM).

The study involved the preparation of 8 formulations of minitablets with BHX and 13 formulations of minitablets containing BFM. Additionally, the SH60 formulation, i.e., containing BFM and 60% of HPMC, was compressed with three different compression forces. All minitablets had a diameter of 3 mm and a target weight of approximately 15 mg. An example of minitablets is presented in Figure 2.

### 3.2. Analysis of Compression Process Parameters

**Figure 3:**
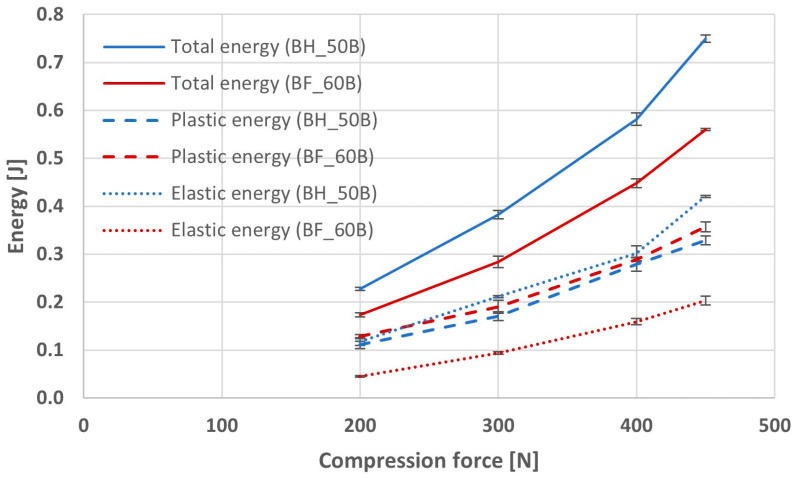
The values of total, plastic, and elastic energies of compression for minitablets with BFM or BHX and 50% or 60% of HPMC with viscosity 4000 cP (Metolose 90SH-4000).

The Trapezium X software (Shimadzu, Kyoto, Japan) was used to calculate the total energy values of the compression process of two selected tablet masses with BHX and BFM compressed with four different compression forces. The software was also used to determine the component of energy responsible for the plastic work of compression, i.e., resulting from the deformation of the particles forming minitablets during the compaction of the powder mass in the die. The elastic component of the compression energy was calculated by subtracting the plastic energy value from the total compression energy. The elastic energy is responsible for the expansion of the tablet mass after the punch stops compressing it. The higher its value, the worse the ability to manufacture tablets with good mechanical properties. The values of total, plastic, and elastic energies obtained in the compression process for the formulation BH_50B and BF_60B are presented in Figure 3.

For each of the series, an increase in the compression force resulted in an increase in the total compression energy as well as its plastic and elastic components. The highest values of these energies were observed in the BH_50B-450N formulation, for which a force of 450 N was used during compression, and the lowest in the BF_60B-200N formulation compressed with a force of 200 N. The total compression energy ranged from 0.17 J to 0.75 J, and its values were much higher in the case of minitablets containing BHX and 50% polymer. Plastic energy values ranged from 0.11 to 0.36 J and were very similar for minitablets with BHX and BFM. The values of elastic energy were again much higher for minitablets containing BHX and 50% polymer than for those with BFM and a higher amount of polymer. Their values ranged from 0.05 J to 0.42 J.

It is also worth noting that the use of a compression force lower than 200 N in the compression process did not lead to formation of tablets. The mechanical resistance of compressed powder was insufficient to carry out further tests. They were damaged or completely disintegrated when they were pushed out of the die.

**Figure 4:**
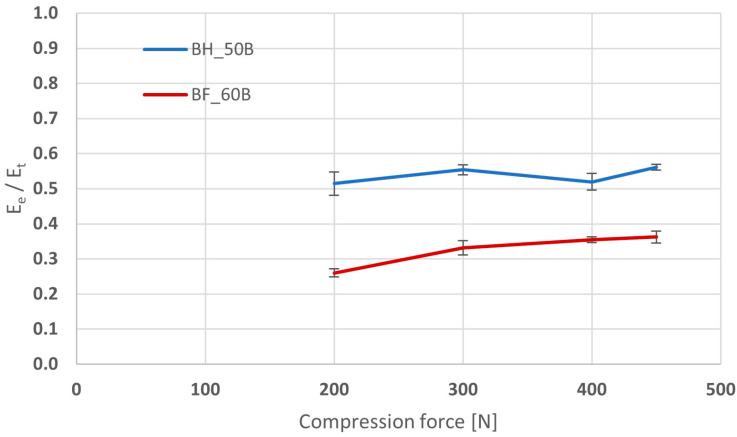
Values of the ratio of elastic energy and total energy depending on the compression force for minitablets with BFM or BHX and 50% or 60% of HPMC with viscosity 4000 cP (Metolose 90SH-4000).

The values of both the total energy and its individual components increased proportionally to a certain extent with the increase in crushing force; therefore, in order to assess the susceptibility of the tablet mass to compression, not were only the values of energy compared but also their mutual ratios. The higher the ratio of elastic energy to plastic energy, the worse the tabletability of the tablet mass and the greater the risk of worsening mechanical parameters with increasing compression force. The values of the ratio of elastic energy to total energy depending on the compression force for the analyzed formulation are shown in Figure 4.

The value of this ratio ranged from 0.51 to 0.56 for BHX minitablets and from 0.26 to 0.36 for BFM minitablets. They increased only slightly with increasing crushing force. The much higher values for the minitablets containing BHX indicate worse compression properties of this active substance, leading to difficulties in achieving satisfactory mechanical properties in the case of this API. The compression properties of HPMC depend on its molecular weight [32]. The higher it is, the poorer the compressibility is because deformation of a longer polymer chain has more elastic character than plastic character. Comparison of values of elastic energy for the BH_50B and BF_60B minitablets did not show such relationships. Even though the amount of HPMC was higher in minitablets with BFM, the values of elastic energy were significantly lower. This may indicate that the compressibility of BFM is much better than that of BHX, and it can even minimize the negative effect of addition of large amount of poorly compressible HPMC.

### 3.3. Hardness of Minitablets

**Figure 5:**
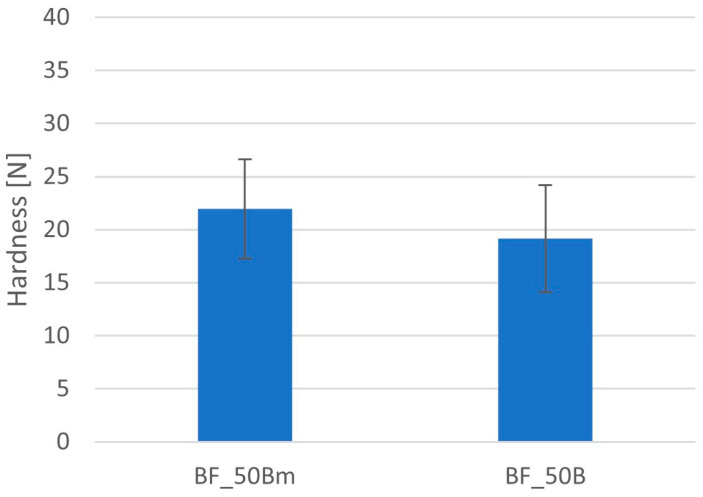
Comparison of the hardness of minitablets with D-mannitol (BF_50Bm) and lactose (BF_50B) containing 50% of HPMC with viscosity 4000 cP (Metolose 90SH-4000).

In order to compare the compressibility of mannitol and spray-dried lactose, two similar series of minitablets containing BFM were made, differing only in the content of these two substances. Minitablets containing mannitol were characterized by a slightly higher hardness of 21.9 ± 4.7 N compared to minitablets containing lactose at 19.1 ± 5.0 N; however, these differences were insignificant and did not exceed standard deviations (Figure 5). Both excipients have similar good compaction properties and are widely used in tablet formulations [33]. Lactose is present in approximately 60% of registered solid oral formulations, while mannitol is present in approximately 20%. The solubility of these two excipients is similar, while low hygroscopicity protects formulations from stability problems. Mannitol has some advantages in orodispersible formulations, such as better mouthfeel and taste and generally faster disintegration, but its disadvantage is the higher price [34]. Due to the fact that there was no clear advantage of either of these two excipients for our formulation, we chose lactose for the preparation of further series of minitablets because its price is lower. Furthermore, the risk of promoting tablet disintegration is lower than in the case of D-mannitol [33,34].

**Figure 6:**
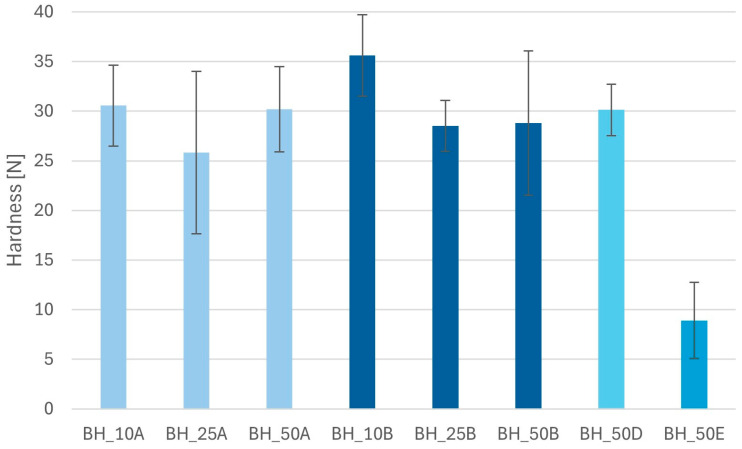
Hardness of the minitablets with BHX and different polymers (A—HPMC 400 cP; B—HPMC 4000 cP; D—sodium alginate; E—PVA).

Minitablets with BHX had a hardness ranging from 8.9 ± 3.8 N to 35.6 ± 4.1 N (Figure 6). The highest value of this parameter was found for minitablets containing 10% Metolose 90SH-4000, while the lowest value was noticed in the case of minitablets with polyvinyl alcohol. Even the lowest value may be considered a good one, considering that the diameter of the tablets was only 3 mm. There was no straight relationship between the polymer content and the hardness of the minitablets found. The standard deviations of the results were relatively high, which might be due to the effect of average flowability.

**Figure 7:**
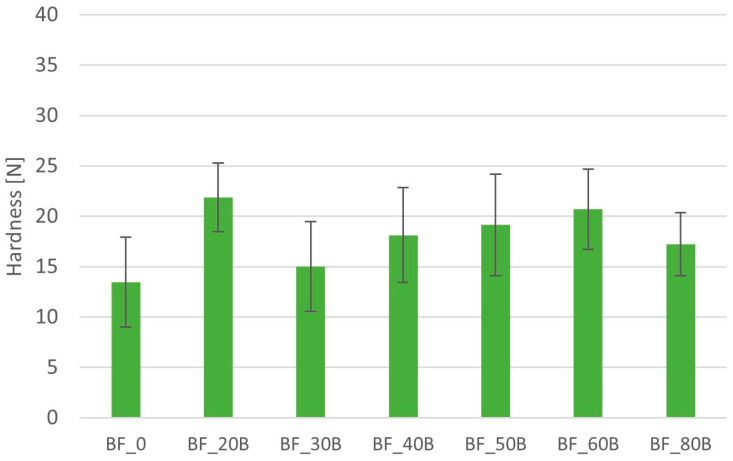
Hardness of the minitablets with BFM and Metolose 90SH-4000.

**Figure 8:**
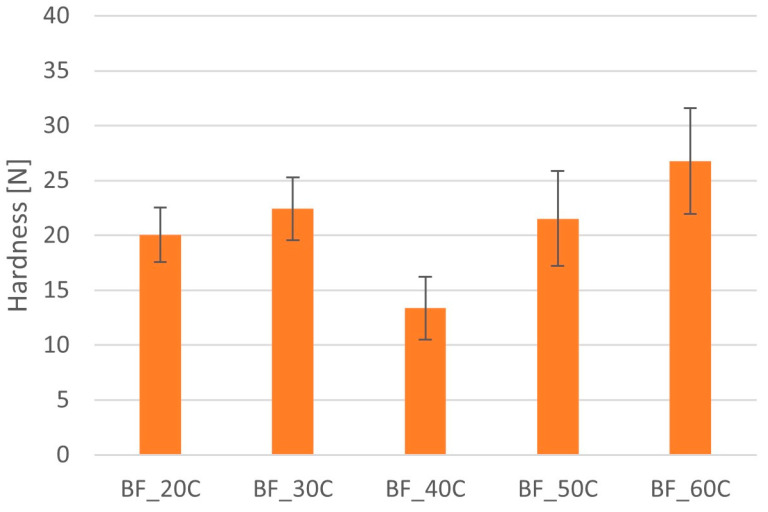
Hardness of minitablets with BFM and Metolose SM-4000.

No relationship was found between the hardness of the minitablets with BFM and the kind of polymer used for the formulation (HPMC or MC) (Figure 7 and Figure 8). Minitablets containing HPMC had a hardness ranging from 13.5 ± 4.4 N to 21.8 ± 3.4 N (Figure 7). There was no relationship between the amount of polymer and the hardness of the minitablets. In this case, tablets with a polymer content of 20% had the highest hardness. In the case of minitablets containing MC, the hardness ranged from 13.4 ± 2.8 N to 26.8 ± 4.8 N (Figure 8). Minitablets containing 60% polymer were characterized by the highest hardness and those containing 40% MC with the lowest hardness.

**Figure 9:**
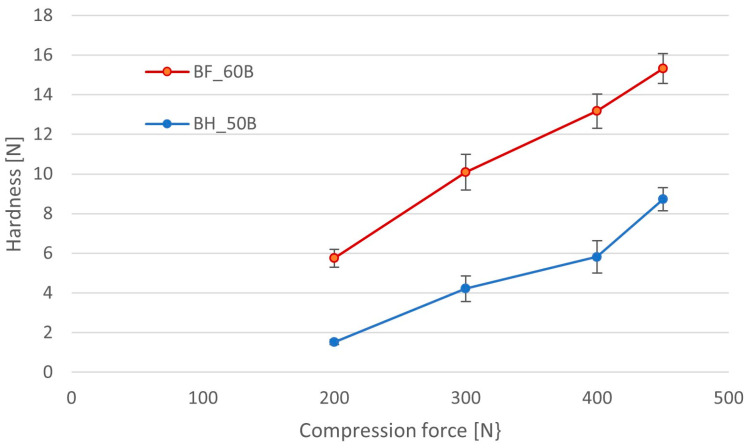
The effect of compression force on the hardness of minitablets with BFM or BHX and 50% or 60% of HPMC with viscosity 4000 cP (Metolose 90SH-4000).

Evaluation of the impact of compression force on the hardness of minitablets, performed using the Shimadzu EZ-SX texture analyzer, showed a proportional increase in the hardness of minitablets with increasing compression pressure (Figure 9). Hardness values were significantly lower for minitablets containing BHX than for those with BFM. Those with BHX had hardness in the range from 1.5 ± 0.1 N to 8.7 ± 0.6 N and those with BFM from 5.7 ± 0.4 N to 15.3 ± 0.8 N. These results correspond very well to the analysis of the elastic and plastic components of compression energy. The ratio of the elastic component of the energy to its total value was much higher in the case of BHX minitablets, indicating its poorer compressibility, which might be the reason for the worse mechanical resistance.

### 3.4. Dissolution Studies

BFM- and BHX-release studies were performed for all batches of prepared minitablets. The studies involved evaluation of the relationship between the release rate of the drug substance and the type and content of the polymer in the formulation as well as the compression force used for the tableting process.

**Figure 10:**
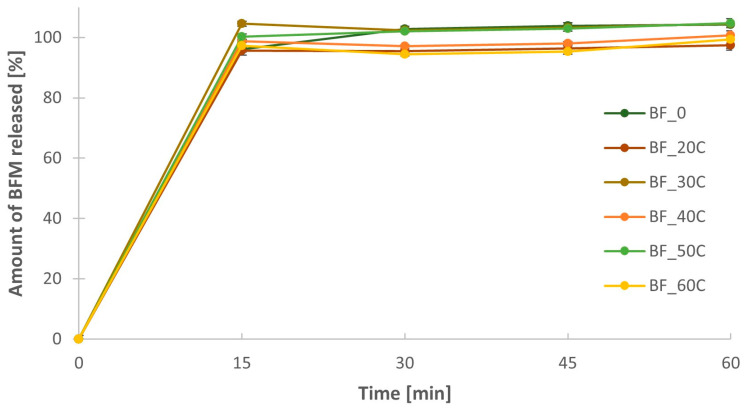
Dissolution profiles for formulations containing BFM and different amount of Metolose SM-4000 (methylcellulose).

In the case of formulations containing Metolose SM, there was no prolonged release of the drug substance from the minitablets in the case of any of the formulations. More than 90% of BFM was released after 15 min from each of them regardless of the amount of polymer in the tablet (Figure 10). The dissolution profiles were similar to the dissolution profile for the formulation BF_0, which was prepared without the addition of polymer as a control series. It is worth mentioning that the standard deviation values for all time points and all formulations with MC were very low. The mean dissolution rate (MDR) for all formulations with MC was similar (Table 4). Its range was from 0.12 to 0.33 h, which was equal to or even lower than in the case of formulation without polymer (BF_0). Furthermore, the values of AUC for the formulations containing MC were also in a similar range as for the formulation without polymer. Their values were from 118.4 to 127.9 µg·h/mL.

**Figure 11:**
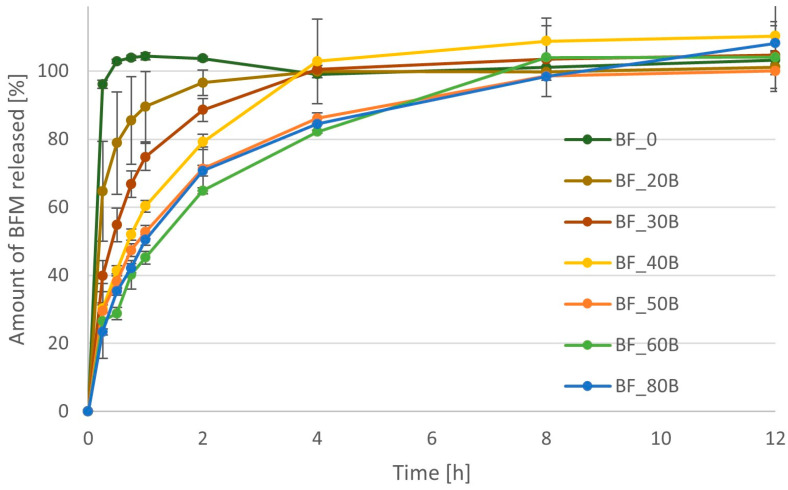
Dissolution profiles for formulations containing BFM and different amount of Metolose 90SH-4000 (hydroxypropylmethylcellulose—HPMC).

Formulations containing HPMC were characterized by different release profiles depending on the polymer content in the minitablets (Figure 11). The time after which 90% of the BFM was released ranged from 1 h for the formulation containing 20% HPMC to as long as 8 h for the formulation comprised 50%, 60%, or 80% of this polymer. Increasing the polymer content in the minitablets in the range of 0–50% resulted in a gradual decrease in the release rate of BFM from the tablets. However, the addition of a higher amount of polymer did not slow the release rate down further, and the dissolution profiles for minitablets comprised 50%, 60%, and 80% of HPMC were very similar. The values of mean dissolution time (MDT) were significantly different for formulations with different amount of HPMC (*p* < 0.05), and their values increased from 0.54 h to 2.52 h when the amount of polymer was increased from 20% to 80% of the tablet mass (Table 4). Values of AUC were significantly lower for the formulations comprised 50% to 80% of the polymer than for the formulations with a lower amount, yet all of them were lower than in the case of minitablets without polymer or those containing MC.

The large standard deviations in some results, especially at the first time points, can be partially explained by the flotation of some minitablets in the dissolution medium. This was particularly noticed in the case of the BF_20B and BF_40B series (Figure 11).

**Figure 12:**
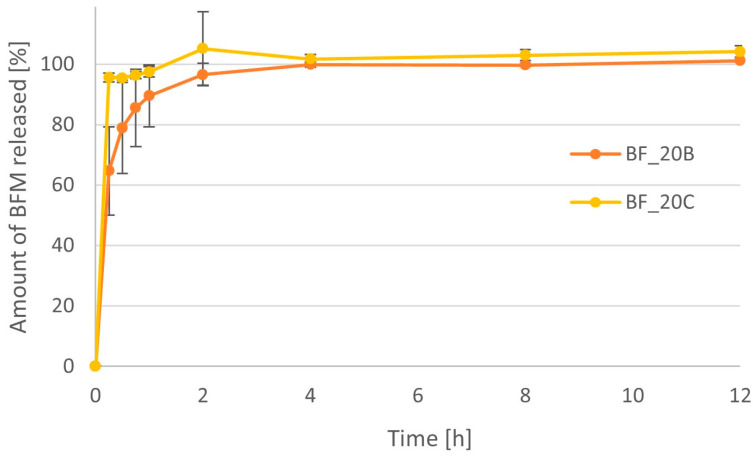
Comparison of the dissolution profiles of BFM from minitablets containing 20% of the HPMC (BF_20B) and 20% of MC (BF_20C) with viscosity 4000 cP.

**Figure 13:**
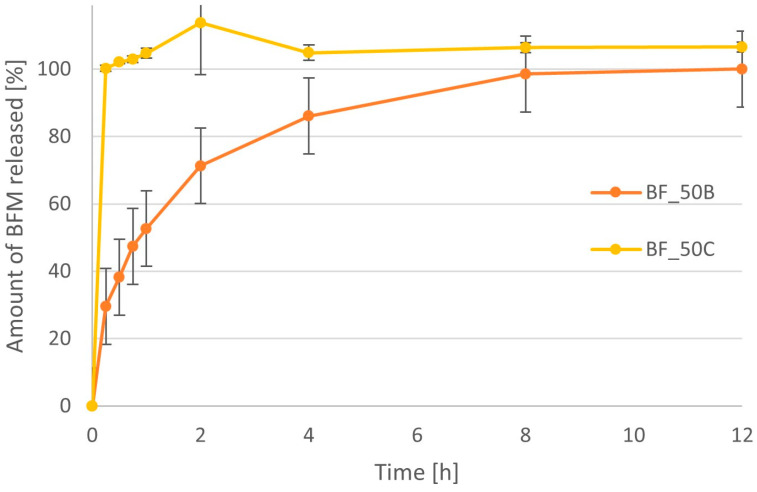
Comparison of the dissolution profiles of BFM from minitablets containing 50% of the HPMC (BF_50B) and MC (BF_50C) with viscosity 4000 cP.

The BFM release profiles for minitablets containing the same amount of polymer but of different types are compared in Figure 12 and Figure 13. The time after which more than 90% of the drug substance was released was approximately 15 min for formulations of 20% MC, while in the case of HPMC, it was approximately 1 h. When 50% polymer was used in the formulations, the differences were even greater. The time needed to achieve 90% drug release was 15 min for MC and approximately 6 h for the HPMC minitablets.

**Figure 14:**
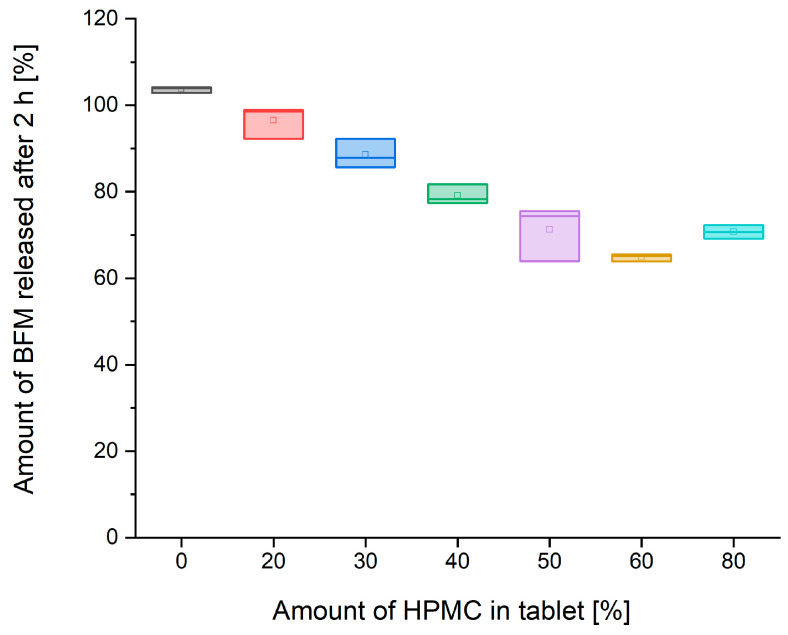
Relationship between the amount of BFM released after 2 h and the amount of Metolose 90SH-4000 (HPMC) in the tablet mass.

**Figure 15:**
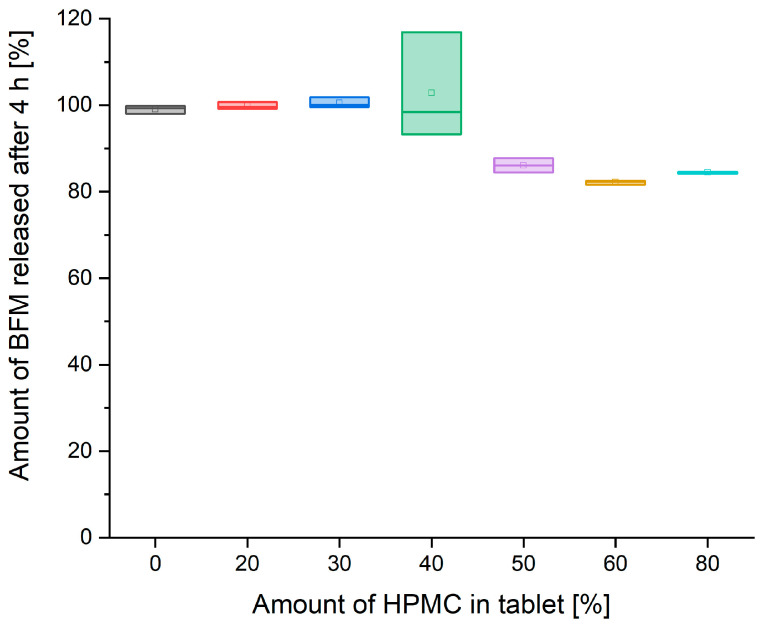
The relationship between the amount of BFM released after 4 h and the amount of HPMC in the tablet mass.

Comparison of drug release rate after 2 and 4 h shows that the influence of the amount of HPMC on the dissolution rate is time-limited (Figure 14 and Figure 15). There was almost a linear decrease in the release rate after 2 h with an increasing amount of HPMC in the tablet mass in the range from 0 to 60%. However, after 4 h of the test, incomplete release was present only in the formulations comprised 50–80% of HPMC, and the amount of released BFM was similar for all formulations having a polymer content within this range.

A similar relationship was found in the studies on metoprolol succinate tablets containing HPMC with viscosity of 100,000 cP [35]. The increase in polymer content in the range of 20–40% led to the elongation of dissolution time. Complete dissolution was achieved after 6 h in the case when the smallest amount of polymer was added and after more than 10 h in the case when 40% HPMC was used. The viscosity of this polymer was much higher than in our study; thus, despite the lower concentration of polymer, a slower dissolution rate was achieved.

The effect of viscosity on the dissolution rate is also dependent on the amount of polymer in the tablet matrix. Studies of Campos-Aldrete [36] showed that it was the highest when the content of HPMC in the tablet was 10%. When it was increased to 20% and 30%, the dissolution rate was similar for three different polymer grades (860 cP, 5000 cP, or 20,000 cP).

**Figure 16:**
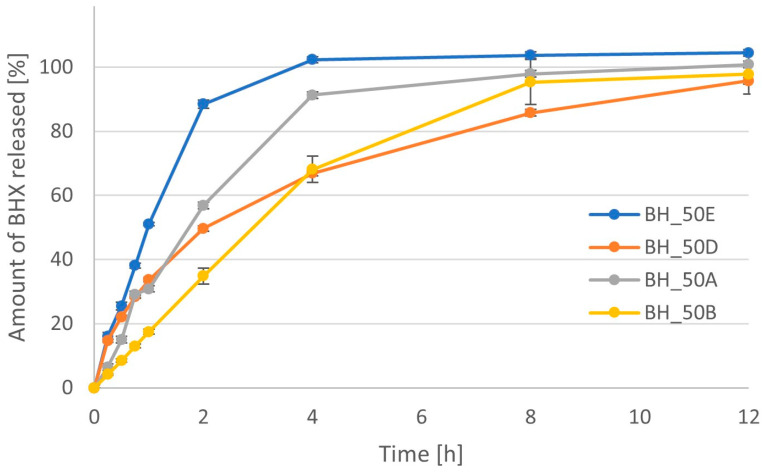
Dissolution profiles for formulations with BHX containing different types of polymer (A—Metolose 90SH-400; B—Metolose 90SH-4000; D—sodium alginate; E—PVA).

In the case of formulations with BHX, four different polymers were used. The slowest dissolution was present in formulations with 50% sodium alginate or HPMC with higher viscosity, where the release of 80% of API took about 8 h (Figure 16). The release from minitablets with Metolose 90SH-4000 cP was slightly slower for the first 4 h, but after that, it was faster than in the case of sodium alginate. The time needed to dissolve 80% of BHX was about 6 h (extrapolated from the curve). The values of mean dissolution rate (MDR) for these two formulations were similar, i.e., 3.30 h for formulation BH_50B and 3.15 h for formulation BH_50D (Table 5). The dissolution of BHX from minitablets containing Metolose with lower viscosity (400 cP) and PVA was much faster. The time needed for the release of more than 80% of API was less than 4 h and 2 h, respectively. MDR for these formulations ranged from 0.32 h to 2.24 h depending on the amount of polymer. The AUC was in the range from 98.5 µg·h/mL to 118.9 µg·h/mL, and its value was significantly lower (*p* < 0.05) only in the case when 50% Metolose 90SH-400 cP was used.

**Figure 17:**
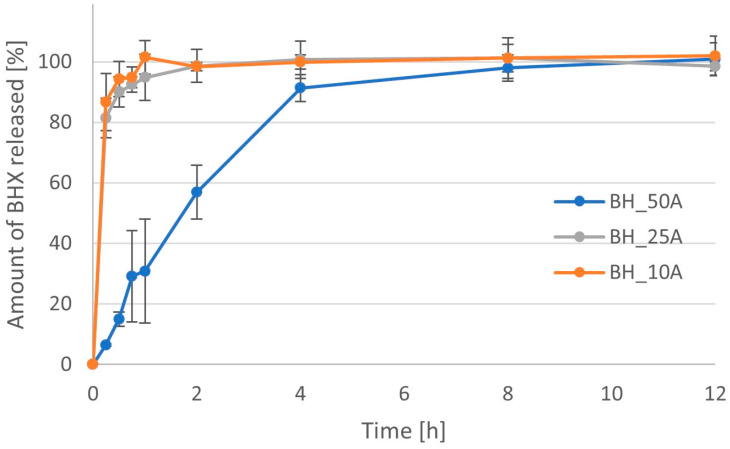
Dissolution profiles for formulations with BHX containing different amount of Metolose 90SH-400 (HPMC with viscosity 400 cP).

**Figure 18:**
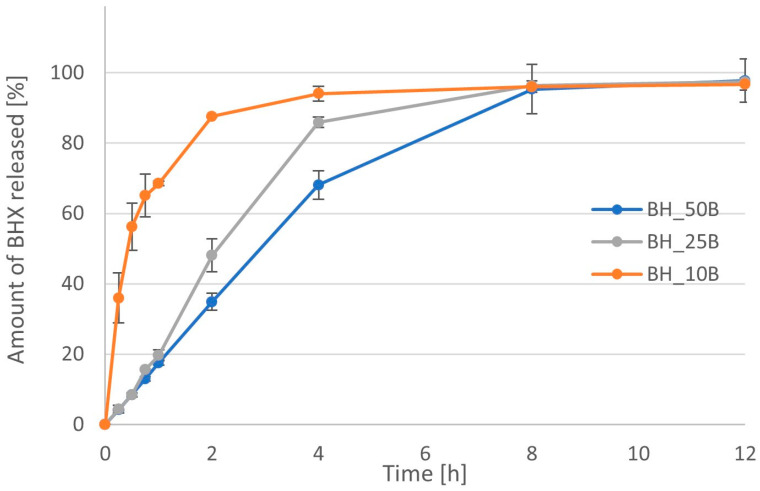
Dissolution profiles for formulations with BHX containing different amount of Metolose 90SH-4000 (HPMC with viscosity 4000 cP).

The effect of different concentrations of Metolose 90SH (HPMC) was clearly visible in the case of both its grades. The higher the amount of polymer, the slower the achieved dissolution. In the case of Metolose with lower viscosity, the addition of 10% and 25% polymer to the tablet mass did not lead to prolonged release of API (Figure 17). MDT was similar for those two formulations, i.e., 0.39 h vs. 0.32 h (Table 5). However, when the amount of polymer was increased to 50% of the tablet mass, the release of more than 80% of API took 4 h. The value of MDT for this formulation was increased to 2.24 h. Using HPMC with higher viscosity led to a much lower dissolution rate (Figure 18, Table 5). Even 10% of this polymer in the formulation slowed a release of 80% of the API at about 2 h. The value of MDT was increased to 0.91 h (as compared with 0.39 h in the case of formulation BH_10A). When the amount of polymer was increased to 25% and 50% of the tablet mass, the time needed for the dissolution of 80% of API was extended to 4 h and 6 h, respectively. The value of the MDT increased in these formulations to 2.46 h and 3.30 h. The area under the dissolution curve decreased significantly when the amount of Metolose 90SH-4000 cP was increased within the range of 10% to 50% of the tablet mass. Its value dropped from 107.3 µg·h/mL to 85.1 µg·h/mL.

As described in other studies, the viscosity of matrix-forming polymer is one of the most important factors affecting dissolution rate. Polymers with higher viscosity form much thicker gel layer on the surface of wetted tablets, which hinders the diffusion of the API outside the tablet. Park et al. [37] found that the gel layer in the case of tablets with ~30% of HPMC of 4000 cP viscosity was 2.5-fold thicker than in the case of tablets with the same amount of low-viscous HPMC. Not only was thickness much higher, but the strength of this hydrogel was also increased when higher-viscous polymer was used, which hindered both the diffusion and erosion of API through this layer. Gao et al. [38] found that the viscosity of the HPMC and its content affected the gel thickness and swelling properties of the matrix tablets. Their studies on the drug release indicated that the major mechanism responsible for drug release was diffusion and not erosion. Therefore, the drug release rate was much higher for low-viscosity HPMC than for HPMC grades with viscosity of 4000 cP–100,000 cP despite a similar thickness of the formed hydrogel layer. HPMC as a soluble polymer is also slowly dissolved during the dissolution. Its viscosity influences the dissolution of the polymer much more than dissolution of API [38]. Interestingly, there are also examples of studies where the viscosity of HPMC had no influence on the release rate of highly soluble API (in the range 4000–100,000 cP), and kinetic studies showed not only the diffusion mechanism of the drug release but also the significant effect of the erosion [39].

Apart from HPMC, we also used sodium alginate and PVA for formulation of minitablets with BHX. Alginates are linear unbranched polysaccharides with different proportions of β-D-mannuronic acid and α-L-guluronic acid residues [40]. They are manufactured in many different grades varying by the ratio of mannuronic to guluronic acid, particle size, and viscosity of the polymer. We used only one grade of sodium alginate with a low viscosity. Despite this, prolonged release was achieved for this formulation in similar extent as in the case of HPMC with a high viscosity. Sodium alginate becomes insoluble in low pH, and the dissolution of BHX was performed with hydrochloric acid solution of pH = 1.2. Therefore, despite the low viscosity of the polymer, the release rate remained low. The PVA that we used in this study (Parteck SRP 80) was also characterized by low viscosity. The advantage of this polymer is the independency of the release rate from pH [41]. However, when used alone, it may not be sufficient to achieve a prolonged release profile [41]. This was the case in our formulation. No prolonged release was present despite the use of 50% PVA in the tablet matrix.

**Figure 19:**
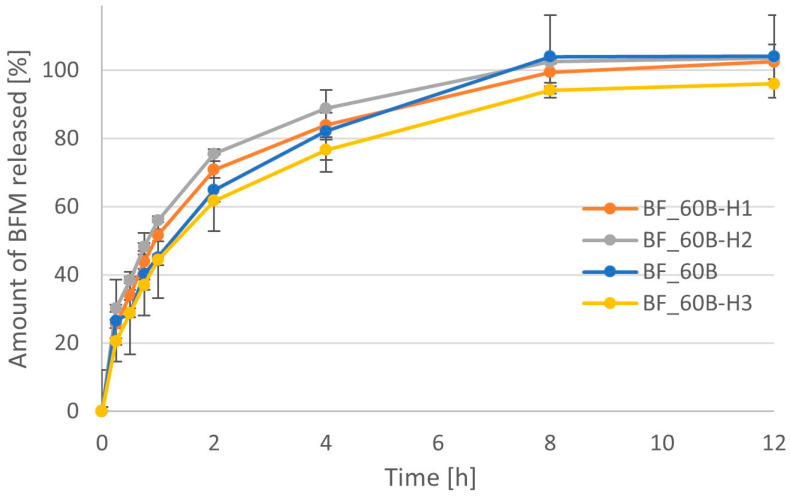
The dissolution profiles of BFM for series of minitablets with a different hardness: <1.5 kp (BF_60B-H1), 1.8–2.0 kp (BF_60B-H2), 2.0–3.0 kp (BF_60B), and 3.0–4.0 kp (BF_60B-H3).

**Figure 20:**
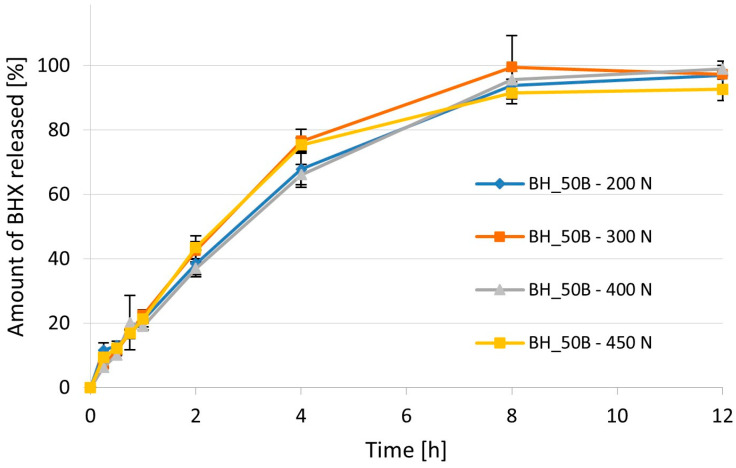
The influence of compression force on the dissolution profile of BHX from minitablets BH_50B.

The effect of compression force on the release of APIs from prepared minitablets was studied in two different ways for BFM and BHX. In the case of BFM, the same tablet press, namely EK0 (Korsch, Berlin, Germany), was used as for the tableting of previously described formulations. It was not equipped with compression force measurement; therefore, the compression force was manually adjusted to achieve minitablets with four different hardness values (<1.5 kp, between 1.8 and 2.0 kp, between 2.0 and 3.0 kp, and from 3.0 to 4.0 kp). The dissolution profiles for these minitablets are presented in Figure 19 In the case of minitablets with BHX, the influence of compression force on the dissolution was studied with minitablets tableted with an EZ-SX texture analyzer (Shimadzu, Suzhou, China) using four different compression forces: 200 N, 300 N, 400 N, and 450 N. The dissolution profiles for these formulations are presented in Figure 20.

There were significant differences (*p* < 0.05) between the amounts of BFM released from minitablets with different hardnesses for the first 4 h. However, after this time, the differences became insignificant. The slowest release of BFM was found in the case of the formulation with the highest hardness, but the fastest was present not in the case of the lowest compression force, as expected, but in the case of tablets with a hardness of 1.8–2.0 kp. In theory, minitablets compressed with a higher compression force have a greater solid fraction and reduced porosity; thus, penetration of water into the tablet matrix should be hampered, and as a consequence, the release of API should be slower. However, despite the significant differences between minitablets with BFM, no clear relationship was found in the effect of compression force on release rate. The values of mean dissolution times were the same (2.20 h) for formulations with hardness 2.0–3.0 kp and 3.0–4.0 kp, but the AUC value was significantly lower for the harder tablets (Table 6).

Analysis of the results of dissolution for minitablets with BHX compressed with different forces showed that in the tested range of compression from 200 N to 450 N, there was no influence of this parameter on the dissolution rate. The significant difference was found only at three time points and only for single pairs of formulations, e.g., 200 N vs. 400 N after 15 and 30 min of the test or 200 N vs. 300 N after 15 min of the test. Therefore, it can be concluded that there was no influence of the compression force on the dissolution rate in the studied formulations. This low significance may be a result of relatively high standard deviations of the results, which might be caused by the flotation of some minitablets during dissolution studies. It is worth mentioning that despite the compression force used, all minitablets from the formulation BH_50B showed prolonged release. The time needed for the dissolution of 80% of BHX was approximately 6 h (value extrapolated from the dissolution curves). Values of mean dissolution time for these formulations were relatively high when compared to other minitablets (Table 7). They ranged from 2.66 to 3.35 h, but a direct relationship between compression force and value of the MDT was not found. Furthermore, values of AUC were not significantly different. They ranged from 85.4 µg·h/mL to 90.9 µg·h/mL.

Studies on the influence of compression force and viscosity of the polymer on the dissolution performed by Khanvilkar et al. [42] showed even lower dependency of the release rate on the compression force used during the tableting process. The influence of this parameter seems to be incomparably lower than the effect of polymer viscosity and content. It may allow the use of higher compression forces for tableting to achieve tablets with better mechanical properties without worries about causing too slow a dissolution rate. However, the limitations of our study on the compression force effect has to be considered. Only four different compression forces were evaluated. This relationship may also be different in the case of different contents of HPMC in the tablet mass. Giunchedi et al. [43] explained that the formation of the gel layer was the first step in the dissolution process, which was not affected by the state of the dry polymer matrix; thus, it was independent of the compression pressure used during the tableting process.

**Figure 21:**
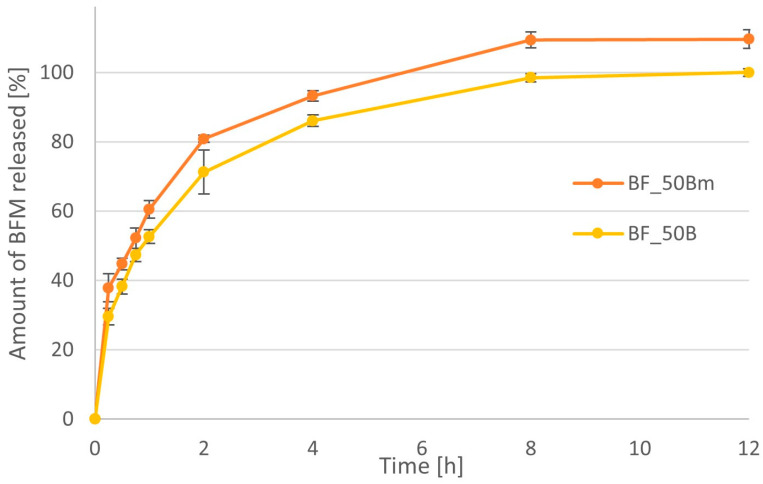
The release profiles of BFM from minitablets containing different fillers: mannitol (BF_50Bm) or lactose (BF_50).

At the beginning of the study, two different fillers were used for the preparation of minitablets, i.e., spray-dried lactose and D-mannitol. Due to insignificant differences in the properties of minitablets with these two excipients, lactose was selected for further studies because we expected that drug release may be slower in the case of this excipient. Although both spray-dried lactose and D-mannitol have similar usefulness in direct compression, similar particle size distribution, flowability, and other physicochemical parameters, including solubility [44], mannitol dissolves faster and is considered as an excipient, which does not slow down dissolution. The comparison of dissolution profiles for two minitablets formulations containing BFM and 50% HPMC with viscosity of 4000 cP confirmed these expectations (Figure 21). The amount of drug release from formulation with mannitol that we compared for the single time point was significantly higher than from the one containing the same amount of lactose (*p* < 0.05). These differences were insignificant only at the 45 min and 2 h time points because the variability of the results was too high (the *p*-value for these points was 0.08 and 0.06). On the other hand, differences between MDT values were not statistically significant. Its value for formulation with mannitol was 1.70 h, while that for the same one containing lactose was 1.79 h.

### 3.5. Uniformity of Minitablets

Testing of the uniformity of tablets is required to ensure that each dosage unit contains the intended amount of active pharmaceutical ingredient (API) and meets specified limits. This test is crucial to maintain consistent therapeutic efficacy and safety. The evaluation of the uniformity of tablets may be based on three different pharmacopeial tests: uniformity of mass [28], uniformity of content [29], and uniformity of dosage units [30]. The uniformity of content test is required when the dose of active substance in the single tablet is lower than 2% or 2 mg, which was the case for the prepared minitablet formulations because they all contained 1 mg of BHX or BFM per single minitablet. These three tests were performed for a randomly selected single batch of minitablets with BHX (BH_50B) and BFM (BF_20C).

The average mass of the minitablets with BHX was 15.40 mg and with BFM was 15.35 mg. The highest deviation was 4.52% and 5.33%, which was below the pharmacopeial limit of ±10%. The content of the APIs also met the pharmacopeial requirements. In the case of the BHX minitablets, the average value was 1.06 mg, and the maximum deviation from the average value was 7.46%. The average BFM content was 1.07 mg, and the maximum deviation from the average value was 7.35%. The pharmacopeial limit according to the monograph 2.9.6 is ±15% for all units and ±25% for not more than 2 out of 20 units.

**Figure 22:**
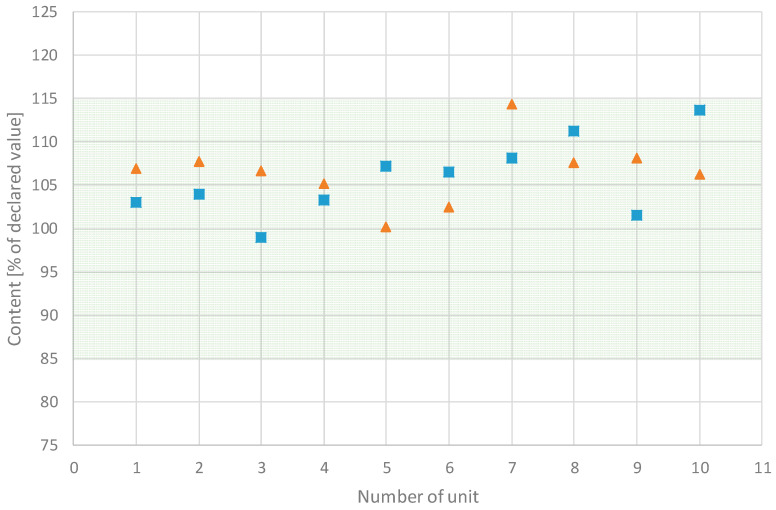
Content of the API in minitablets with BHX (squares) and BFM (triangles). The green area indicates ±15% of the declared value; pink area indicates 75–85% and 115–125% of the declared value.

Monograph 2.9.40 of the European Pharmacopoeia requires comparison of the content in a single tablet unit with declared values rather than an average content of drug substance in the tablet. It requires calculation of the so-called acceptance value (AV). In the case of analyzed minitablets, it should not exceed 15, or if it does, the deviation of a single content from the average value must not exceed 25%. The calculated value of AV was 22.44 for the BHX minitablets and 23.99 for the BFM minitablets, which is above 15 and means that the second criterion must be applied. This was achieved since the maximum deviation for BHX minitablets was 12.16% and for BFM minitablets was 12.91%. Therefore, the prepared minitablets complied with Ph. Eur. 2.9.40 monograph. Figure 22 shows the content of APIs in the analyzed minitablets in relation to declared values.

### 3.6. Kinetic Analysis of the Release Process

The complete results of the kinetic analysis of the release profiles of BFM and BHX from minitablets showing prolonged release are presented in Tables S1 and S2. For all BHX formulations used for the analysis, it was found that either Hopfenberg or Hixson–Crowell models are the best data-fitting equations. This suggests that whenever there is any control of the drug release employed by the formulation, it is based on the erosion of the whole system.

A very different situation was found for BF formulations. In the case of formulations with HPMC (4000 cP) content of 20%, 30%, and 80% (BF_20B, BF_30B, and BF_80B, respectively), the Hopfenberg model was found to be the most predictive one for the dissolution profiles. However, for HPMC content of 40%, 50%, and 60%, the Korsmeyer–Peppas model was the best-fitting one, suggesting a switch to the drug release mechanism based more on diffusion than erosion. These findings are just mere suggestions of drug release mechanisms, yet they pose a valuable asset in the optimization of the formulation. It seems that there might be found an optimal content of HPMC for exhibiting an diffusion-driven control of the drug release based on the well-known and discussed mechanism of the hydrogel barrier, of which thickness and viscosity are the controlling factors of the drug release rate.

We investigated this suggested diffusion controlled drug release mechanism for BF_40B, BF_50B, and BF_60B formulations by cutting down the release profiles to the time points with release below 60%. This is recommended for easy interpretation of the “n” release exponent. We found almost perfect Fickian diffusion for the BF_40B formulation (in fact Higuchi equation), whereas lower values of “n” exponent suggested drug mass transport of a different nature (Table 8) [45]. Focusing on Fickian diffusion for the BF_40B formulation, it could be concluded that it is an example of the controlled release behavior with a well-defined and quantified drug release mechanism and rate. Such findings are certainly an extension beyond the standard prolongation of drug release towards a controlled release system and a starting point for future research.

The differences in the release mechanism between BHX and BFM may be associated with a lower solubility of the BHX. The tablet matrix needed to be eroded in order to release BHX, while freely soluble BFM was released mostly by a diffusion mechanism. Only in the case when the amount of matrix-forming polymer was higher did the release kinetic studies indicate stronger correlations with models based on the erosion. The higher concentration of highly viscous polymer may result in a microenvironment with a very high viscosity, forming a gel layer impermeable even to very soluble BFM. Therefore, in this case, erosion of the matrix was also necessary prior to the release of this API.

## 4. Conclusions

The results of our work proved the possibility of the formulation of prolonged-release minitablets with bromhexine hydrochloride and bisoprolol fumarate. The dose of 1 mg per one minitablet allows easy dose adjustment for pediatric patients of different ages and body weights, while prolonged release of drug substances for up to 8 h allows a single dose administration per day. Due to the lack of prolonged release dosage forms dedicated to children, compliance is significant issue in pediatric pharmacotherapy. The development of minitablets provides the potential to deal with this important problem. Furthermore, formulated minitablets allow easy dose adjustment not only for the pediatric patients but also for adults who need to have their dose decreased, e.g., in the cases of lower body weight (anorexia or cachexia), increased side effects, etc.

The prepared minitablets were characterized by good mechanical properties. Their uniformity was confirmed according to the pharmacopeial requirements, which is crucial for the safety and efficacy of all pharmaceutical products, especially those containing low doses of drug substances, which usually applies to minitablets.

The selection of the appropriate type of polymer and its concentration in a tablet mass led to the prolonged release of BHX and BFM for a predefined time. Furthermore, studies on the kinetics of release mechanisms also showed dependence not only on the type but also on the amount of polymer in a tablet mass.

## Figures and Tables

**Table 1:** Composition of minitablets with bromhexine hydrochloride (BHX).

Amount of Ingredient in Tablet Mass [%]								
Ingredient	BH_10A	BH_25A	BH_50A	BH_10B	BH_25B	BH_50B	BH_50D	BH_50E
BHX	6.67	6.67	6.67	6.67	6.67	6.67	6.67	6.67
Metolose 90SH-400	10.00	25.00	50.00	-	-	-	-	-
Metolose 90SH-4000	-	-	-	10.00	25.00	50.00	-	-
Sodium alginate	-	-	-	-	-	-	50.00	-
PVA	-	-	-	-	-	-	-	50.00
MCC	40.67	33.17	20.67	40.67	33.17	20.67	20.67	20.67
Flowlac	40.67	33.17	20.67	40.67	33.17	20.67	20.67	20.67
Pruv	2.00	2.00	2.00	2.00	2.00	2.00	2.00	2.00

**Table 2:** Composition of minitablets with bisoprolol fumarate (BFM) and Metolose 90SH-4000.

Amount of Ingredient in Tablet Mass [%]								
Ingredient	BF_0	BF_20B	BF_30B	BF_40B	BF_50B	BF_60B	BF_80B	BF_50Bm
BFM	6.67	6.67	6.67	6.67	6.67	6.67	6.67	6.67
Metolose 90SH-4000	0.00	20.00	30.00	40.00	50.00	60.00	80.00	50.00
MCC	44.67	34.67	29.33	24.67	19.33	14.67	4.67	19.33
Flowlac	44.67	34.67	30.00	24.67	20.00	14.67	4.67	-
Pearlitol	-	-	-	-	-	-	-	20.00
Cab-O-Sil	2.00	2.00	2.00	2.00	2.00	2.00	2.00	2.00
Pruv	2.00	2.00	2.00	2.00	2.00	2.00	2.00	2.00

**Table 3:** Composition of minitablets with bisoprolol fumarate (BFM) and Metolose SM-4000.

Amount of Ingredient in Tablet Mass [%]					
Ingredient	BF_20C	BF_30C	BF_40C	BF_50C	BF_60C
BFM	6.67	6.67	6.67	6.67	6.67
Metolose SM-4000	20.00	30.00	40.00	50.00	60.00
MCC	34.67	29.33	24.67	19.33	14.67
Flowlac	34.67	30.00	24.67	20.00	14.67
Cab-O-Sil	2.00	2.00	2.00	2.00	2.00
Pruv	2.00	2.00	2.00	2.00	2.00

**Table 4:** Dissolution parameters for BFM minitablets (MDT—mean dissolution time; AUC—area under the dissolution curve).

Formulation	MDT [h]	AUC [µg·h/mL]
BF_0	0.33	120.4
BF_20B	0.54	115.9
BF_30B	1.06	114.5
BF_40B	1.56	115.1
BF_50B	1.79	102.1
BF_60B	2.20	102.0
BF_80B	2.52	102.6
BF_50Bm	1.70	113.0
BF_20C	0.33	121.6
BF_30C	0.28	127.9
BF_40C	0.15	119.7
BF_50C	0.12	126.6
BF_60C	0.16	118.4

**Table 5:** Dissolution parameters for BHX minitablets (MDT—mean dissolution time; AUC—area under the dissolution curve).

Formulation	MDT [h]	AUC [µg·h/mL]
BH_10A	0.39	118.9
BH_25A	0.32	117.7
BH_50A	2.24	98.5
BH_10B	0.91	107.3
BH_25B	2.46	93.0
BH_50B	3.30	85.1
BH_50D	3.15	84.7
BH_50E	1.32	111.6

**Table 6:** Dissolution parameters for BFM minitablets compressed with different forces (MDT—mean dissolution time; AUC—area under the dissolution curve).

Formulation	MDT [h]	AUC [µg·h/mL]
BF_60B-H1	2.06	101.9
BF_60B-H2	1.76	106.1
BF_60B	2.20	102.0
BF_60B-H3	2.20	94.1

**Table 7:** Dissolution parameters for BHX minitablets compressed with different forces (MDT—mean dissolution time; AUC—area under the dissolution curve).

Formulation	MDT [h]	AUC [µg·h/mL]
BH_50B—200N	3.19	85.4
BH_50B—300N	2.89	90.9
BH_50B—400N	3.35	85.5
BH_50B—450N	2.66	86.6

**Table 8:** Korsmeyer–Peppas equation (Q = K × t^n^) parameters for BFM prolonged-release formulations.

Formulation	K	n
BF_40B	60.04	0.51
BF_50B	44.38	0.44
BF_60B	52.78	0.43

## Data Availability

Dataset available from the authors on request.

## Supplementary Materials

The following supporting information can be downloaded at https://www.mdpi.com/article/10.3390/pharmaceutics16091153/s1, Table S1: Results of the kinetic modeling for minitablets containing BHX. Table S2: Results of the kinetic modeling for minitablets containing BFM.
