# Supplementary material for: Application of Hydrophilic Polymers to the Preparation of Prolonged-Release Minitablets with Bromhexine Hydrochloride and Bisoprolol Fumarate
Source: Pharmaceutics. 2024 Aug 30;16(9):1153. doi: 10.3390/pharmaceutics16091153 (PMC11434728; doi:10.3390/pharmaceutics16091153)
Supplement: Supplementary file 1 [file pharmaceutics-16-01153-s001.zip › pharmaceutics-3150301-supplementary.pdf]

pharmaceutics

MDPI

Supplementary Material

Application of Hydrophilic Polymers to the Preparation of Prolonged-Release Minitablets with Bromhexine Hydrochloride and Bisoprolol Fumarate

Agata Grzejdziak [1], Witold Brniak [2,*], Olaf Lengier [1], Justyna Anna Żarek [1], Dziyana Hliabovich [1] and Aleksander Mendyk [2]

[1] Students Scientific Group of Pharmaceutical Technology, Faculty of Pharmacy, Jagiellonian University Medical College, Medyczna 9, 30-688 Kraków, Poland
[2] Department of Pharmaceutical Technology and Biopharmaceutics, Faculty of Pharmacy, Jagiellonian University Medical College, Medyczna 9, 30-688 Kraków, Poland
* Correspondence: w.brniak@uj.edu.pl

Table S1. Results of the kinetic modeling for minitablets containing BHX (colors indicate RMSE value range: green - < 5.00; yellow – 5.00 - 10.00; red > 10.00; bold – the lowest value for the formulation).

Model name	BH_10B	BH_25B	BH_50A	BH_50B	BH_50D	BH_50E
Zero order	34.45	15.02	26.12	7.94	23.99	18.07
First order	5.67	6.99	4.18	4.99	5.42	6.19
Second order	2.34	9.79	8.41	8.59	5.18	10.76
Third order	5.48	14.13	13.31	12.26	9.36	14.67
Higuchi	13.67	12.65	11.78	10.94	6.89	9.12
Hixson-Crowley	9.83	**5.19**	**3.76**	2.62	8.11	**3.53**
Korsmeyer-Peppas	5.22	9.92	11.56	4.57	4.19	7.99
Hopfenberg	**1.90**	6.99	4.18	**1.83**	**3.53**	6.19

Table S2. Results of kinetic modeling for minitablets containing BFM (colors indicate RMSE value range: green - < 5.00; yellow – 5.00 - 10.00; red > 10.00; bold – the lowest value for the formulation).

Model name	BF_20B	BF_30B	BF_40B	BF_50B	BF_60B	BF_80B
Zero order	38.70	35.24	24.43	25.54	25.54	32.80
First order	5.82	4.59	5.05	6.64	6.64	5.80
Second order	1.09	3.86	6.84	6.86	6.86	6.27
Third order	4.64	7.51	9.79	10.24	10.24	10.19
Higuchi	17.64	13.37	4.34	6.04	6.04	12.61
Hixson-Crowley	11.35	9.20	7.11	8.75	8.75	12.40
Korsmeyer-Peppas	2.52	4.25	**1.69**	**2.94**	**2.94**	5.19
Hopfenberg	**0.75**	**2.37**	4.61	5.57	5.57	**4.44**
